# Brain-wide representation of social knowledge

**DOI:** 10.1093/scan/nsae032

**Published:** 2024-06-13

**Authors:** Daniel Alcalá-López, Ning Mei, Pedro Margolles, David Soto

**Affiliations:** Consciousness group, Basque Center on Cognition, Brain and Language, San Sebastian 20009, Spain; Psychology Department, Shenzhen University, Nanshan district, Guangdong province 3688, China; Consciousness group, Basque Center on Cognition, Brain and Language, San Sebastian 20009, Spain; Consciousness group, Basque Center on Cognition, Brain and Language, San Sebastian 20009, Spain

**Keywords:** social cognition, abstract concepts, language models, searchlight decoding

## Abstract

Understanding how the human brain maps different dimensions of social conceptualizations remains a key unresolved issue. We performed a functional magnetic resonance imaging (MRI) study in which participants were exposed to audio definitions of personality traits and asked to simulate experiences associated with the concepts. Half of the concepts were affective (e.g. empathetic), and the other half were non-affective (e.g. intelligent). Orthogonally, half of the concepts were highly likable (e.g. sincere) and half were socially undesirable (e.g. liar). Behaviourally, we observed that the dimension of social desirability reflected the participant’s subjective ratings better than affect. FMRI decoding results showed that both social desirability and affect could be decoded in local patterns of activity through distributed brain regions including the superior temporal, inferior frontal, precuneus and key nodes of the default mode network in posterior/anterior cingulate and ventromedial prefrontal cortex. Decoding accuracy was better for social desirability than affect. A representational similarity analysis further demonstrated that a deep language model significantly predicted brain activity associated with the concepts in bilateral regions of superior and anterior temporal lobes. The results demonstrate a brain-wide representation of social knowledge, involving default model network systems that support the multimodal simulation of social experience, with a further reliance on language-related preprocessing.

## Introduction

The past two decades have witnessed a flourishing interest in understanding how the human brain represents semantic knowledge (Martin, 2007; Bauer and Just, 2019). Early functional MRI studies (Haxby *et al.*, 2021; Mitchell *et al.*, 2003) showed that brain activity patterns in a set of brain regions, usually referred to as the semantic network, carry information about the concept that the observer is experiencing (e.g. animals vs tools). The semantic network comprises areas in the superior and inferior temporal lobes and parietal, inferior frontal and medial prefrontal cortex (Binder *et al.*, 2009). These findings have sparked a lively discussion on the nature of semantic representations. Originally, theoretical models suggested that the brain represents concepts as amodal symbols (Fodor, 1975). A more recent approach argues that conceptual representations are grounded in the sensorimotor processes associated with them (Barsalou, 1999; Prinz, 2004) re-enacted via mental simulation (Soto *et al.*, 2020). This grounded cognition framework was initially conceived for the study of concrete concepts. Only recently, there has been a similar attempt at studying the representations of abstract concepts. Unlike concrete concepts, these are not perceptually bound to a physical object as referent. Hence, abstract concepts are likely grounded beyond pure sensorimotor systems (Shea, 2018), including more complex representations of events or situations that can only rely on perceptual and action systems to a limited extent (Wilson-Mendenhall *et al.*, 2011). This view is congruent with a recent two-systems proposal of semantic representation by Borghi *et al.* (2019). These authors argue that, although a sensorimotor feature-based system would be common for the representation of both concrete and abstract concepts, the latter would need the assistance of an additional system that incorporates more complex linguistic and social information (Borghi *et al.*, 2019; Fini *et al.*, 2021; Fini *et al.*, 2022).

Prior studies using mass-univariate functional magnetic resonance imaging (fMRI) approaches showed brain areas with overlapping activation during the presentation of abstract and concrete concepts, including key areas of the semantic network (Binder *et al.*, 2005). However, more recent attempts to study the neural representations of abstract concepts using multivariate pattern analyses have shown that higher-order regions in the frontal cortex are involved in the representation of abstract relative to concrete concepts. For instance, Ghio *et al.* (2016) showed that abstract *vs.* concrete concepts (e.g. emotional or mathematical v*s.* action concepts) can be decoded from brain activity patterns in the inferior frontal gyrus and the insula. This study further highlighted that fine-grained representations of conceptual categories appear to co-exist along the concrete-to-abstract continuum (e.g. number, emotion, moral, aesthetic or social concepts).

Despite this promising research, current understanding of how the human brain represents social knowledge is still at an early stage of development. Psychological research suggests that people represent information about others across several dimensions (i.e. social *vs.* nonsocial states, emotion and agency). Perhaps one of the more robust findings is the involvement of the anterior temporal lobe. Mass-univariate studies (Zahn *et al.*, 2007; Pobric *et al.*, 2016; Binney *et al.*, 2016; Lin *et al.*, 2018) have found activity increases in this brain region when participants are presented with different sources of information regarding other individuals, and a recent fMRI study combining both uni- and multivariate approaches observed that information related to people can be decoded from the anterior temporal lobe (Wang *et al.*, 2017). The medial prefrontal cortex has also been implicated in the representation of psychological traits. In an fMRI study, Ma *et al.* (2014) asked participants to infer the psychological traits of other individuals from a series of descriptions. First, a sentence with an implicit psychological trait was presented. Then, a second target sentence appeared and participants had to infer the individual’s trait, which could be either congruent or not with the first sentence. They found that activity patterns within the ventral medial prefrontal cortex showed a neural adaptation effect indicative of the representation of trait knowledge: when the target psychological trait was congruent with the prior implicit sentence, neural activation decreased faster than during incongruent psychological trait descriptions (Ma *et al.*, 2014). Hassabis *et al.* (2014) used multivariate pattern analysis to show that mental imagery contents regarding personality traits such as agreeableness and extraversion can be decoded from the medial prefrontal cortex. In addition, moral reasoning based on narratives regarding intentional *vs.* accidental harm (Koster-Hale *et al.*, 2013) and also emotional states conveyed through verbal descriptions (Skerry and Saxe, 2015) or facial information (Skerry and Saxe, 2014) can be decoded from core regions of the mentalizing network (Carrington and Bailey, 2009) including the temporoparietal junction and dorsomedial prefrontal cortex.

Affect is a fundamental feature underlying many psychosocial phenomena (Barrett and Bliss-Moreau, 2009). However, a large tradition of psychological research has emphasized the extent to which human judgements of one’s own and others’ experiences are deeply influenced by their likableness or social desirability (Anderson, 1968; Fisher *et al.*, 1985), indicating that this may be a key underlying feature of the representation of social concepts. Nevertheless, it is not yet clear how the brain represents these different aspects of social information. This is the main goal of the present study. On the one hand, we assessed how the brain represents the affective content of social concepts by contrasting concepts related to the affective traits of other people, such as cruel or caring and concepts that refer to non-affective traits, such as selfish or intelligent. On the other hand, we assessed the representation of social desirability by comparing highly likable concepts, such as empathetic or understanding, to socially undesirable concepts, such as phony or insensitive. Finally, we used a computational approach in which the representations of a language model (GPT2) fed with our concept definitions was used to perform a representational similarity analysis (RSA) (Kriegeskorte *et al.*, 2008) within an encoding framework (Konkle and Alvarez, 2022), to map the brain representation of social concepts. RSA and encoding models have been previously used to explain brain responses to concrete concepts (Devereux *et al.*, 2013; Anderson *et al.*, 2015; Martin *et al.*, 2018; Mitchell *et al.*, 2008), and recent research has shown similarities in the representations of deep language transformer models and the brain responses during speech (Caucheteux and King, 2022). Here, we used a similar approach to understand the brain representation of abstract social concepts and test the contribution of language-related representations.

## Methods

### Participants

We scanned 30 participants (mean age 24.07 ± 3.67 years; 18 females). The sample size was selected based on related fMRI studies of social cognition [Koster-Hale *et al.* (2013), *N* = 23; Tamir *et al.* (2016), *N* = 20] and abstract concepts [Ghio *et al.* (2016), *N* = 36; Skerry and Saxe (2014), *N* = 22]. Participants had normal or corrected-to-normal vision, gave written informed consent prior to the experiment and were financially compensated with 20 euros for their participation. The experiment lasted for about an hour and a half and was approved by the BCBL Ethics Review Board in compliance with the Declaration of Helsinki.

### MRI acquisition

The present fMRI study was performed on a SIEMENS’s Magnetom Prisma-fit scanner with a 3T magnet and a 64-channel head coil. We collected one high-resolution T1-weighted image and eight functional runs for each participant. Each functional run consisted of a multiband gradient echo-planar imaging sequence with an acceleration factor of 6, a resolution of 2.4 x 2.4 x 2.4 mm^3^, a repetition time of 850 ms, a echo time of 35 ms and a bandwidth of 2582 Hz/Px, which was used to obtain 537 3D volumes of the whole brain (66 slices; field of view (FOV = 210 mm).

The auditory stimuli for the experimental task (i.e. the concept definitions) were presented through earphones (S14, Sensimetrics, Malden, MA). Presentation volume was adjusted to a comfortable level for each participant. The visual elements of the experimental setup (e.g. fixation cross) were projected on an MRI-compatible, out-of-bore screen using a projector in the adjacent room.

### Experimental procedure

**Fig. 1.**
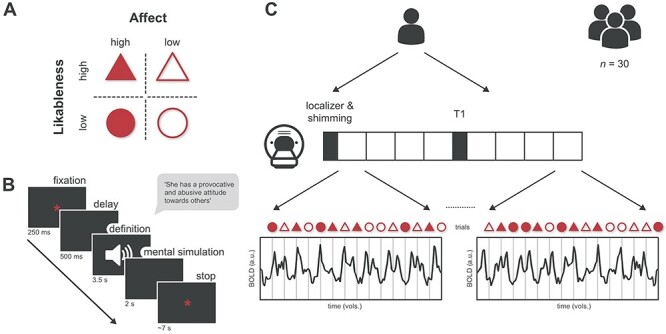
Illustration of the experiment workflow with sub-figures labelled from A to C, (A) A total of 36 social concept definitions matched one of our four subcategories reflecting a combination of the affect and social desirability of the social knowledge. (B) Participants listened to the definition of a social concept and were asked to mentally simulate a person behaving the way described in the definition. (C) We acquired one anatomical and eight functional sequences in a single scanning session.

We selected 36 social concepts from the list of 555 personality trait words used in the study of Anderson (1968) to assess and rank the words based on the likability ratings in college students. We developed short audio definitions referring to the 36 social concepts controlling for sentence length. We also analyzed the average frequency of the items within each definition using Espal (Duchon *et al.*, 2013), and this was similar across the different pairs of affective and social desirability conditions (lowest *P* value = 0.432; Desirable/High Affect: *M* = 9410.73, STD = 3890.79; Desirable/Low Affect: Mean (M) = 8075.72, Standard deviation (STD) = 3684.09; Undesirable/High Affect: *M* = 8075.72, STD = 3684.09; Undesirable/Low Affect: *M* = 8485.76, STD = 2795.76). We categorized all social concepts following a 2×2 factorial design using the concept dimensions of affect and social desirability. First, half of the concepts were affective, making an explicit mention to the emotions of oneself or others (see the left panel in Table 1), while the other half involved non-affective, referring to interpersonal behavior that does not explicitly involve any emotional content or state (see the right panel in Table 1). Second, half of the concepts involved socially desirable interpersonal behavior (see the upper half in Table 1), whereas the other half described social undesirable behavior (see the bottom half in Table 1). We kept the number of concepts in each category equivalent, with nine social concepts in each of the four subcategories (e.g. high affect and low social desirability). Each trial began with a fixation period of 250 ms followed by a blank screen for 500 ms (Figure 1B). Then, participants listened to the definition of a social concept for 3500 ms (e.g. ‘She gets sad when seeing someone suffering and tries to ease their pain’; see Table 1 for the complete list of social concept definitions), followed by another period of 2000 ms in which they were instructed to mentally simulate a person of their own choice (e.g. a relative, acquaintance or famous character) behaving as described in the definition. The above were only examples given to participants in order to encourage them to think about the concepts.

**Table 1.** Definitions of social concepts

Affective-state concepts			Mental-state concepts	
Concept	Definition	Likableness	Concept	Definition
Empathetic	‘She puts herself on someone else’s shoes and feels in her own flesh how they feel’	High	Sincere	‘She says what she really thinks, without lying or pretending’.
Good-natured	‘Her way of being shows sympathy, simplicity and kindness’.	High	Understanding	‘She understands the reason behind others’ behavior and is tolerant’.
Kind	‘She naturally tends to behave well and do good to others’.	High	Loyal	‘She always acts with respect and fidelity to her commitments or to others’.
Cheerful	‘She conveys her cheerful and pleasant character’.	High	Intelligent	‘She can reason, solve problems and understand complex ideas’.
Warm-hearted	‘She treats others with affection and desires to be in company’.	High	Unselfish	‘She is inclined to give and share with others beyond her own interest’.
Enthusiastic	‘She tends to get very excited easily almost all the time’.	High	Clever	‘She has the ability to invent things by combining intelligence and skill’.
Grateful	‘She values very much and is very happy when someone does her a favor’.	High	Helpful	‘She enjoys doing things and combining efforts with others’.
Sensible	‘She gets excited at displays of feelings such as love or compassion’.	High	Forgiving	‘She tends to forgive offenses and doesn’t judge others harshly’.
Sympathetic	‘She is saddened when she sees someone suffering and tries to ease their suffering’.	High	Conscientious	‘She puts a lot of attention and care into everything she does’.
Cruel	‘She does not feel compassion for or take pleasure in the suffering of others’.	Low	Phony	‘She’s pretending to be someone she’s not to fool others’.
Insensitive	‘She neither thrills nor perceives the feelings of others’.	Low	Greedy	‘She always tries to accumulate more and Greedy more stuff, and never shares with anyone else’.
Snobbish	‘When she speaks, she makes others feel despised’.	Low	Rude	‘She has no manners and speaks without respect for others’.
Unforgiving	‘She forgives no one and shows no compassion’.	Low	Selfish	‘She is not interested in the interests of others, only in her own convenience’.
Gloomy	‘She always despairs because she can onlv see the negative side of things’.	Low	Hostile	‘She has a provocative and abusive attitude towards others’.
Resentful	‘She behaves as if life is treating her badly all the time’.	Low	Boring	‘She annoys others with her lack of fun or interest in things’.
Neurotic	‘She is very unstable and reacts to things in an emotional and exaggerated way’.	Low	Prejudiced	‘She judges others based on negative preconceptions’.
Hot-tempered	‘She loses her temper easily and reacts aggressively to others’.	Low	Irresponsible	‘She is unaware of her obligations and acts without foresight’.
Envious	‘She feels sad or angry when she doesn’t have what other people have’.	Low	Lazy	‘She never carries out the tasks she should’.

All concepts used in the experiment followed a 2×2 factorial design. Half of the concepts made an explicit mention to the emotions, while the other half referred to interpersonal behavior that does not explicitly involve any emotional content or state. Second, half of the concepts involved socially desirable behavior, whereas the other half were socially undesirable.

All 36 social concepts were presented in each functional run, with concept order randomized between runs. A run lasted approximately six and a half minutes. To facilitate the estimation of the peak of the hemodynamic response function (HRF) across the different trials, we included an additional jitter so that the time between the offset of the current stimulus and the onset of the next audio definition varied between 6 and 8 s. The jitter followed a pseudo-exponential distribution resulting in 50% of trials with an intertrial interval of 6 s, 25% of 6.5 s, 12.5% of 7 s and so on. All experimental procedures for stimulus delivery during the mental simulation task were programmed and presented using PsychoPy v.1.83.0.4 (Peirce, 2007).

### Rating task

Before and after the MRI scanning session, we asked participants to rate the affect and social desirability of each concept definition on a scale from 0 to 100. We used these measurements to analyze the test–retest reliability of self-ratings of the concept definitions.

### MRI data preprocessing

We first converted all MRI data from DICOM to NIfTI format using MRIConvert (http://lcni.uoregon.edu/downloads/mriconvert). We then preprocessed the MRI data using FEAT 6 (fMRI Expert Analysis Tool) from the FMRIB Software Library (FSL suite; v5.0.9). We removed the first 10 volumes of each functional run to ensure steady-state magnetization. We used FSL’s brain extraction tool 2.1 to remove non-brain tissue (Smith, 2002) and Automatic Removal of Motion Artifacts to identify and remove motion-related artifacts (Pruim *et al.*, 2015). We applied spatial smoothing to the data using a Gaussian kernel of 3 mm full width half minimum and a high-pass filter with a cutoff of 90 s. All functional images were coaligned to a reference volume from the first run for each participant.

#### Data preparation

After preprocessing the MRI data, we used the output generated from PsychoPy during the experimental task to label the relevant scans with an attribute for each class (i.e. high *vs.* low affect; high *vs.* low social desirability). We then removed invariant features (i.e. voxels whose BOLD activity did not vary throughout the length of a functional run) and stacked the data from all eight functional runs after *z*-score normalization and linear detrending (Figure 1D). Finally, we generated examples for the multivoxel pattern analysis (MVPA) analysis by averaging BOLD signals between 5.5 and 10.5 s after stimulus onset. Given that the audio definitions lasted for about 3.5 s, this timeframe was selected to ensure that our BOLD examples for classification contained information from the peak of the HRF associated with processing the content of the definition.

### Whole-brain searchlight multivariate pattern analysis

We conducted a whole-brain searchlight multivariate pattern analysis [whole-brain searchlight MVPA, Haxby *et al.* (2021); Kriegeskorte *et al.* (2006)], implemented in the Python libraries scikit-learn (Pedregosa *et al.*, 2011) and nilearn (Estève, 2015). The searchlight algorithm used a sphere with a 4-mm radius. The voxel values of each sphere were vectorized and used as features to predict the affect (high *vs.* low affect) or social desirability (high *vs.* low social desirability) of the concepts. This was achieved by cross-validating a linear support vector machine classifier [(SVC) (Suthaharan, 2016)]. The SVC was implemented by scikit-learn (Pedregosa *et al.*, 2011) using the default hyperparameters. The SVC was nested with a calibration stage to provide probabilistic predictions of the classifications (Niculescu-Mizil and Caruana, 2005; Wilks, 1990). Prior to feeding the features to the SVC, we first standardized the features by mean-centering and reducing the variance to 1. This standardization was performed in the training set and then applied to the test set. Within each searchlight sphere, we first split the data, which contained a matrix of features (n_examples × n_voxels) and a vector of labels (n_examples, being either high *vs.* low affect or high *vs.* low social desirability), into training and testing sets by a 80–20% ratio. This stratified cross-validation scheme did not take into account the run. A further analysis using a leave-one-run out cross-validation scheme was conducted to mitigate the possibility of any carry-over effects across trials influencing the decoding scores. We further performed a leave-two-concepts out crossvalidation approach to further test the generalizability of the representations; any cross-trial data leakage is expected to be negligible here since only one trial of each class in each run was used for testing.

We measured the classification accuracy of the SVC on the test set using the area under the receiver operating characteristic curve (ROC AUC). This was repeated 100 times to estimate the variance of the cross-validation performance. The average performance of the cross-validation was assigned to the center of the searchlight sphere. The searchlight was performed for each subject in native space and separately for affect and social desirability dimensions. We conducted a similar decoding experiment but replaced the SVC by a dummy classifier,^1^ which ignored the features/voxels and randomly predicted the labels. The dummy classifier was also nested within the standardization step as described earlier. The ROC AUC scores of the dummy classifier were used for estimating the chance level decoding scores. We then subtracted the chance level decoding scores from the corresponding decoding scores. The brain maps were then normalized to the standard space using FSL tools and fed to the FSL randomize algorithm (Jenkinson *et al.*, 2012) with the threshold-free cluster enhancement (Smith and Nichols, 2009) clustering enhancement to find clusters in which decoding was greater than zero, which indicated that the decoding scores were greater than the chance level scores. The number of permutations was 10 000.

### Decoding social dimensions from GPT2 model representations

We used the GPT2 natural language processing (NLP) transformer model (Radford *et al.*, 2019) to extract features from the sentences used in the experiment. GPT2 is one of the state-of-the-art large NLP models trained using very large corpus datasets. GPT2 provides excellent transfer learning performance in translation, text generalization and summarization.

Each of the 36 sentences was tokenized and fed to the Spanish GPT2 model ^2^ provided by HuggingFace [Wolf *et al.* (2019)^3^]. The representations were extracted from the eighth layer activation of the GPT2 model. According to Caucheteux *et al.* (2022), sixth to ninth layers of GPT-2 best predict brain activity, and the study showed that eighth was the best among the layers. The maximum token size was 21. The middle layer representation dimension for each token was 768. Thus, the flattened feature representation of each sentence had 16 128 elements.^4^ In other words, each sentence was represented by 16 128 length vectors. We then conducted a decoding analysis on the GPT2 model (Radford *et al.*, 2019) representations of the stimulus sentences separately for affect and likableness. The analysis aimed to decode whether the sentences were associated with low or high affect/likableness.

Decoding analysis was quantified by a leave-a-pair-of-words-out cross-validation procedure. For instance, in affect condition, one low-affect word and one high-affect word were left out as the testing data, while the rest were used for training a linear SVC (i.e. low *vs.* high affect). Then, the statistical significance was measured by means of a permutation test. During the permutation test, the correspondence between the features and the labels were shuffled, and the same cross-validation was performed, so that the average decoding score was used as an estimate of the empirical chance level. The permutation procedure was repeated 1000 times to estimate the distribution of the empirical chance level. The significance was measured by the probability of the empirical chance level being greater than the average of actual decoding scores. The cross-validation^5^ and permutation test^6^ were conducted using Scikit-learn (Pedregosa *et al.*, 2011; Abraham *et al.*, 2014).

### Standard RSA

After the representations of the 36 sentences were extracted from the GPT2 model (Radford *et al.*, 2019; Wolf *et al.*, 2019), the representational dissimilarity matrix (RDM) of the features of the sentences was computed using 1 – Pearson correlation implemented by Scipy [Virtanen *et al.* (2020)^7^]. The RDM was then used for further model-based RSA analyses of the fMRI data. The fMRI data were averaged for each sentence. The resulting data matrix per participant had a shape of 36 by *n*_voxels). We then extracted voxels using a moving sphere with radius of 4 mm. The RDM of the averaged voxel data was computed using 1 – Pearson correlation implemented by Scipy (Virtanen *et al.*, 2020). We then correlated the GPT2 model RDM and the fMRI RDM using Spearman correlation using a searchlight approach. The correlation coefficient was assigned to the center of the sphere. This resulted in a whole-brain map of RSA correlation coefficients for each subject. These were normalized by using the Fisher inverse hyperbolic tangent transform. In order to estimate the significant clusters of the RSA maps across subjects, we conducted the same RSA procedure but with shuffled fMRI data. This allowed us to estimate the empirical chance level of the RSA maps. The difference between the whole-brain RSA maps and the chance level RSA maps was then computed. RSA was conducted in native space. The RSA maps were then transformed to standard space and fed to the FSL randomise algorithm (Jenkinson *et al.*, 2012) to perform statistical inference at the group level. Threshold-free cluster enhancement (Smith and Nichols, 2009) was used to find spatial clusters of RSA maps significantly greater than zero. The number of permutations was 10 000.

### Encoding-based RSA

Konkle and Alvarez (2022) proposed an encoding-based RSA pipeline, adding encoding models on top of the standard RSA pipeline. This procedure helps to contextualize better the information patterns from the GPT2 model space into the brain space. Because of the large number of features per sentence (i.e. 16 128) in the GPT2 model, it is important to increase the number of examples to overcome any overfitting problem in the encoding model. Hence, the data were split into train and test sets by leaving one of the participants out. Encoding-based RSA analysis was performed in standard space. A L2-regularized linear regression model (Ridge regression) implemented in Scikit-learn (Hilt and Seegrist, 1977; Pedregosa *et al.*, 2011) was applied to the training set and then used to predict the voxel values using the GPT2 middle layer representation of the sentences. Ridge regression was nested in a grid search algorithm to cross-validate the best L2-regularization term by leaving-one-subject-out within the training set. The trained ridge regression model predicted the voxel values in the test set. The one-dimensional GPT2 feature vector was mapped to the flattened fMRI voxel values. The regression coefficient matrix was too big for our computer RAM. Thus, the fMRI voxel values were divided into 20 sets. This means that 20 independent ridge regression models were trained and they were used to predict each small set of the voxel values in the test set. The predicted voxel value matrices were averaged for each sentence and concatenated for further analysis. The brain RDM of the predicted voxels from the encoding model correlated with the brain RDM of the fMRI activity of the left-out subject. This was performed by using Spearman correlation within each sphere of a searchlight moving across the brain. A map of correlation coefficients was generated for each subject left as the test set. We conducted the same RSA procedures but with the shuffled fMRI data to estimate the chance level encoding RSA maps. The randomize procedure was applied to the difference between the encoding-based RSA maps and the chance level RSA maps, as described earlier.

### Noise ceiling analysis

We also computed the noise ceiling (Nili *et al.*, 2014) regarding the RSA. The fMRI data were converted to standard space. Within each searchlight sphere, each subject’s data were averaged across the different trials of each social concept. A representational dissimilarity matrix (36 by 36) was computed within each subject. On each iteration of the analysis, we compared each subject’s distance matrix relative to the mean of the remaining subjects, which produced lower noise ceiling estimates of all the subjects. The average of these estimates represented the lower noise ceiling. Additionally, we also compared each subject’s distance matrix relative to the average of all subjects, including the left-out subject, in order to determine the upper noise ceiling estimates (Nili *et al.*, 2014). The lower noise ceilings and the upper noise ceilings were then assigned to the center of each searchlight sphere. The process is repeated through the whole brain in order to provide a noise ceiling map.

### Visualization

Brain map visualizations were made in the cortical surface by using the Freesurfer surface mesh geometry.^8^ The reference surface mesh geometry was the Freesurfer fsaverage surface (Fischl *et al.*, 1999),^9^ and the transformation was linearly interpolated by a spatial window of 3 mm. For the correlation maps, any values smaller than $1e^{-3}$ were not shown.

## Results

### Behavioral results

**Fig. 2.**
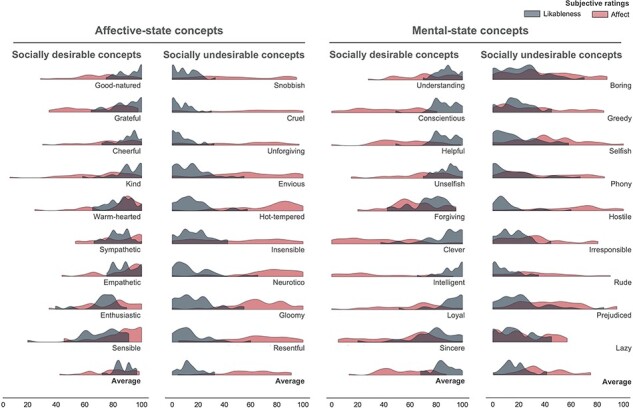
Distributions of ratings of social concepts. Participants read each concept definition and rated the extent to which the described behavior involved the emotions of oneself or others (affect; *red*) as well as whether such behavior was socially desirable (social desirability; *gray*) on a scale from 0 (very non-affective; very unlikable) to 100 (very affective; very likable).

Subjective ratings of the concept definitions showed that participants categorized social concepts in terms of their affect and social desirability as expected based on their normative definitions (Anderson (1968); Figure 2). A paired *t*-test confirmed that ratings of affect among the affective concepts (*M* = 68.514, stantard deviation (SD) = 12.614) were significantly higher than the non-affective (*M* = 45.813, SD = 13.442; *t*$_{(29)}$ = 8.026, *P* < 0.001, *d* = 1.465, logBF10 = 14.35). Similarly, ratings of social desirability among the concepts defined as socially desirable (*M* = 84.472, SD = 6.580) were significantly higher than those selected as highly unlikable (*M* = 15.413, SD = 8.285; *t*$_{(29)}$ = 30.382, *P* < 0.001, *d* = 5.547, logBF10 = 46.69).

Such a difference between ratings of high *vs* low affect (*M* = 22.701, SD = 15.493) was smaller than the difference between ratings of high *vs* low social desirability (*M* = 69.060, SD = 12.450; *t*$_{(539)}$ = -13.925, *P* < 0.001, *d* = –2.542, logBF10 = 26.42). Note that a logBF10 greater than 2.2 is considered an overwhelming support in favor of the alternative hypothesis (Kass and Raftery, 1995). This suggests that the social desirability of others’ behavior is more salient for the representation of social knowledge than affect. This is congruent with the results from the test–retest repeatability analysis. The intraclass correlation coefficient (ICC) showed that the reliability of the ratings before and after the scanning session was fair for affect [ICC = 0.47; 95% confidence interval (0.36–0.60)] and excellent for social desirability [ICC = 0.93; 95% confidence interval (0.89–0.96)].

### Whole-brain searchlight classification analyses

**Fig. 3.**
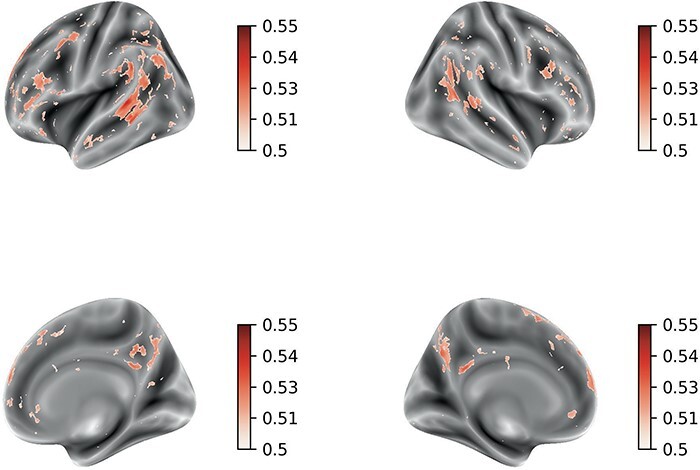
NeuAverage whole-brain searchlight classification scores of the affect dimension (affective *vs* non-affective) of the social concepts. The heatmap levels represent the clusters where the ROC-AUC scores were statistically significant.

**Fig. 4.**
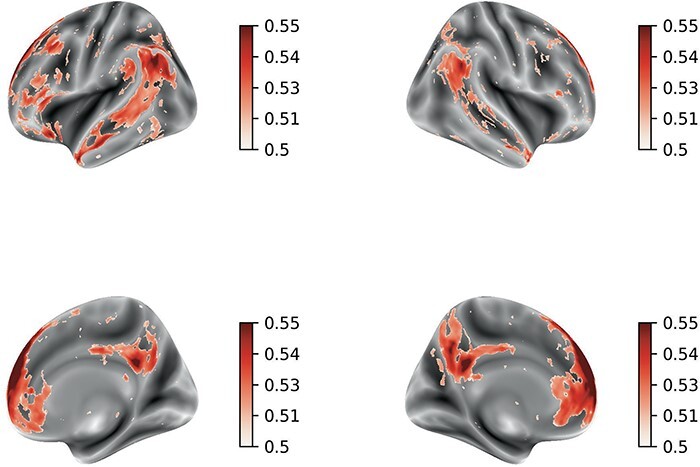
Neuroimaging results. Average whole-brain searchlight classification scores of social desirability. The heatmap levels represent the clusters where the ROC-AUC scores were statistically significant.

**Fig. 5.**
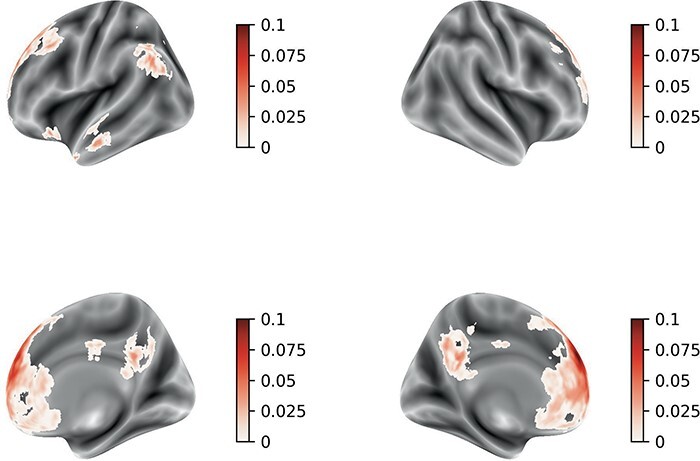
Neuroimaging results. Average difference in the whole-brain searchlight ROC-AUC scores where decoding social desirability was better than decoding affect. The heatmap levels represent clusters that were statistically significant.

First, we conducted whole-brain searchlight classification analyses to decode (i) the affect and (ii) the social desirability of the auditory definitions presented to participants. Figure 3 and 4 show significant clusters in a distributed network of bilateral regions in which both social desirability and affect were decoded. Social desirability could be decoded in superior, middle and anterior temporal cortex; anterior and posterior cingulate and precuneus; and also in dorsolateral, ventrolateral and dorsomedial anterior prefrontal areas. Affect was also decoded from multivoxel patterns in similar areas. Then, we compared the decoding scores between the two searchlights. and observed that decoding of social desirability was better compared to affect Figure 5. There were no significant clusters in which decoding was better for affect compared to social desirability.

Similar searchlight classification maps were found with different cross-validation procedures (i) using a leave-one-run out (Supplementary Figures 1, 2 and 3) and (ii) in a procedure in which a pair of words were left out for testing the classifier (Supplementary Figures 4, 5 and 6).

### Representation similarity analysis

We conducted the standard RSA (Kriegeskorte *et al.*, 2008) and also encoding-based RSA (Konkle and Alvarez, 2022) (see the Methods section) to understand how a language model (i.e. GPT2) of the sentences for the different social concepts explained the brain responses.

First, we report the results of a decoding analysis in which the hidden layer of the language model was used to predict the social desirability and the affect dimensions of the concepts (see the Methods section). Decoding accuracy indexed by the ROC-AUC was significantly above chance (i.e. decoding affect: ROC AUC = 0.7369 ± 0.1699, *µ* ± *σ*, *p* < 0.05; decoding social desirability: ROC AUC = 0.6238 ± 0.1846, *µ* ± *σ*, *P* < 0.05), indicating that the model representations contain information that is predictive of these social dimensions. These results are depicted in Supplementary Figures 7 and 8.

**Fig. 6.**
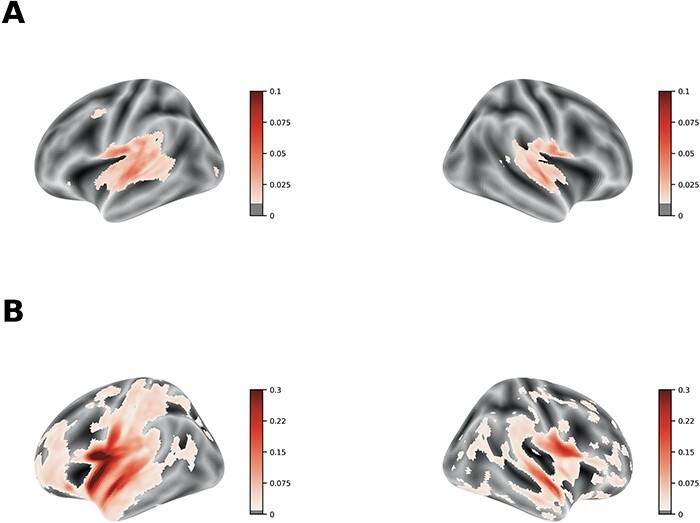
Neuroimaging results. Average correlation coefficient maps and the corresponding corrected *P* value maps of the standard RSA and encoding-based RSA. (A) The correlation coefficients of the standard RSA that were greater than the empirical chance level. The average correlation maps were masked by the randomized P value cluster map that thresholded voxels with a significance level of 0.05. (B) The clusters where the average correlation coefficients of the encoding-based RSA were greater than the empirical chance level.

The standard RSA results revealed significant associations between the language model and the brain responses, notably, around the Heshchl gyrus, planun temporale and superior temporal areas bilaterally further extending into the anterior temporal lobe (Figure 6).

We furthermore performed a noise ceiling analysis regarding the RSA (Nili *et al.*, 2014). Noise ceiling maps were computed across the whole brain using a searchlight approach (see the Methods section). The noise ceiling represents the maximum similarity score that could be achieved given the noise level inherent in the data. Taking the noise ceiling into account, the clusters found in the temporal lobe in the standard RSA fell below the lower bound of the noise ceiling, while the clusters found in the encoding-based RSA were greater than the lower bound of the noise ceiling, but lower than the upper bound (Supplementary Figure 9).

## Discussion

The present fMRI study investigated how social knowledge related to mental state concepts associated with personality traits is represented in the human brain. Searchlight decoding analyses showed that affective and social desirability dimensions of the concepts can be decoded from a brain wide distributed network of regions including both anterior and posterior cingulate and precuneus, middle, superior and anterior temporal cortex; posterior parietal cortex; temporoparietal junction; and ventromedial and lateral prefrontal cortex. Notably, decoding of social desirability in many of these regions was higher relative to affect. This result is consonant with the participants’ self-reports. Subjective ratings of social desirability associated with the auditory definitions of the concepts were more concentrated in the extreme values of the distribution for corresponding high and low normative values of social desirability according to a previous study (Anderson, 1968). Moreover, these subjective ratings of social desirability were consistent with recent replication studies (Dumas *et al.*, 2002; Chandler, 2018).

Model-based representational similarity analyses showed that brain activity patterns in language related areas in temporal cortices bilaterally encoded the representation of the concepts extracted from a language model (GPT2). Recent research has shown that the representations of large language models such as GPT2 map linearly onto the brain responses of participants listening to stories (Caucheteux and King, 2022; Caucheteux *et al.*, 2023). Deep language models such as GPT2 are trained to predict words from their context in the sentences, and, in principle, the representations of these models seem unlikely to capture the social meaning of the sentences. However, our decoding analysis based on the hidden representation of the language models indicated that the representations of GPT2 were informative to some extent of the affective and likableness dimensions of the definitions presented to participants. Therefore, the model-based RSA results likely reflect a language-like or compositional representation of the concepts that bears to some extent on the dimensions of likableness and affect, but that otherwise disregards the personal, experiential, multimodal nature of mental simulations of social concepts which is supported by regions in the default-mode network. We also note that the superior temporal cortex has also been implicated in social perception based on auditory (Belin *et al.*, 2000; Kriegstein and Giraud, 2004) and visual cues (Zilbovicius *et al.*, 2006; Allison *et al.*, 2000) and also in theory of mind (Deen *et al.*, 2015; Schultz *et al.*, 2004; Gallagher *et al.*, 2000; Heekeren *et al.*, 2003). Therefore, the decoding- and encoding-based RSA results observed in this superior temporal cortical substrates likely reflect a combination of both language-based representations and more specific representations of social dimensions related to likableness and affect.

Previous studies showed the involvement of the anterior cingulate cortex in the detection of positively valenced attributes during social evaluation tasks, related both to the self (Sharot *et al.*, 2007) and also other people (Hughes and Beer, 2012). Additional studies indicated that anterior cingulate cortex is implicated in the detection of valence and the reporting of rewarding attributes during social evaluation (Rigney *et al.*, 2017), thereby playing a role in processing salient cues related to the self (Perini *et al.*, 2018). Our results suggest that the anterior cingulate cortex, as part of the salience network (Uddin, 2015), represents distinct aspects of social knowledge related to the social desirability and affect dimensions, with social desirability receiving a higher weight. Notably, this pattern of results was observed in a task context that did not require participants to perform overt responses to external stimuli in a social setting, but rather required mental simulation of social situations associated with the auditory definitions based on personal, idiosyncratic experiences.

Although the anterior temporal lobe has received much attention in recent years due to its involvement in the processing of abstract concepts (Binney *et al.*, 2016; Hoffman *et al.*, 2015; Wang *et al.*, 2017), our results do not place this brain region in a privileged position regarding the representation of the social concepts. While social desirability could be decoded in anterior temporal cortex, a brain wide distributed network of regions was implicated, including putative areas of a social cognition system for theory of mind in the posterior cingulate and precuneus, the temporoparietal junction and dorsal and ventral medial prefrontal cortex (Ma *et al.*, 2014; Alcalá-López *et al.*, 2018; Adolphs, 1999) as well as canonical language regions.

Tamir and colleagues used fMRI in conjunction with representational similarity analyses to delineate how the brain represents internal states of other individuals (Tamir *et al.*, 2016). Participants had to consider up to 60 different internal states (e.g. drunkenness or satisfaction). Then, RSA was used to explain fMRI responses based on a theoretical model of how different social dimensions inter-relate when subjects perform a matching task based on two visual scenarios potentially associated with a given concept (e.g. ‘awe’). The results showed that three concept dimensions, namely, rationality, social impact and valence, explained a significant amount of variance in brain responses associated with other people’s mental states (Tamir *et al.* (2016); see also Thornton and Tamir (2020); Thornton and Mitchell (2018)). The present results expand on this prior work to define the contribution of social desirability and affect dimensions of social knowledge, while also revealing the contribution from language representations. Concerning the differences in the number of concept dimensions between the present study and the study by Tamir *et al.* (2016), it is possible that the likableness or social desirability dimension identified here is related to the social impact dimension identified by Tamir and colleagues (2016), which also involved significant clusters of brain activity predicted by the model in similar brain regions to those identified here by the decoding and encoding RSA results. However, the RSA results from the study by Tamir and colleagues (2016) showed that the valence dimension was represented in a left-lateralized set of regions including the dorsolateral and ventrolateral prefrontal cortex and the temporoparietal junction. This result is in contrast to the present study in which affective valence was decoded from corresponding bilateral areas, also implicating additional temporal regions and posterior cingulate and precuneus cortex. Also, the RSA based on a language model explained brain activity patterns in a bilateral network.

In sum, the results from the current study underscore the brain wide, distributed nature of social knowledge representations which rely on the interplay between language systems and default-mode network systems that support the personal mental simulations of social conceptualizations. This observation is in line with recent studies demonstrating the involvement of both domain-specific and heteromodal cortical regions in the representation of concrete concepts (Tong *et al.*, 2022). It should be noted that social concepts, like emotional concepts, represent subclusters of abstract conceptualizations (Villani *et al.*, 2019). In addition to social concepts there are philosophical, spiritual, physical, spatiotemporal, and quantitative concepts. Future studies can further test the brain representation of abstract knowledge by assessing a wider range of abstract conceptualizations within the same experimental procedure.

## Supplementary Material

nsae032_Supp

## Data Availability

The analysis scripts can be found at https://github.com/nmningmei/. The experimental data will be uploaded to OpenNeuro upon acceptance for publication.

## Footnotes

1
https://scikit-learn.org/stable/modules/generated/sklearn.dummy.DummyClassifier.html

2 The pretrained Spanish GPT2 model https://huggingface.co/DeepESP/gpt2-spanish

3 Using the Huggingface interface (https://github.com/huggingface/transformers)

4 Details of feature extraction is described in https://tinyurl.com/ye2av23y

5 sklearn.model_selection.cross_validate: https://tinyurl.com/5ezx234w

6 sklearn.model_selection.permutation_test_score: https://tinyurl.com/bddz7s4t

7 Scipy.spatial.distance.pdist: https://tinyurl.com/yz75v5f9

8 nilearn.surface.vol$\_$to$\_$surf: https://tinyurl.com/ykmprdtz

9 nilearn.datasets.fetch_surf_fsaverage:https://tinyurl.com/4xuau39r
