# Supplementary figures and images for: Brain-wide representation of social knowledge
Source: Soc Cogn Affect Neurosci. 2024 Jun 13;19(1):nsae032. doi: 10.1093/scan/nsae032 (PMC11173195; doi:10.1093/scan/nsae032)

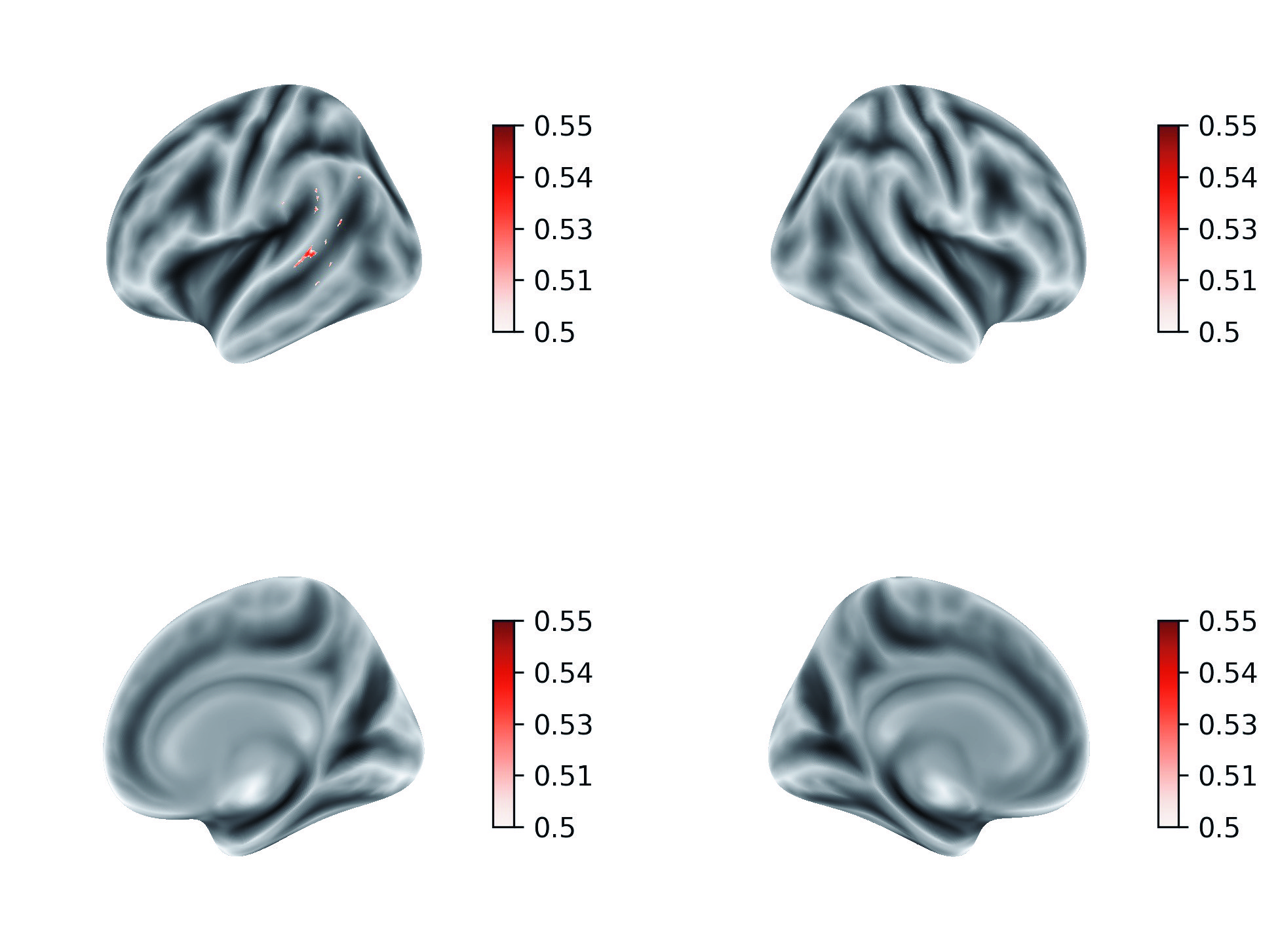

Supplement: nsae032_Supp [file nsae032_supp.zip › sFig1.jpg]

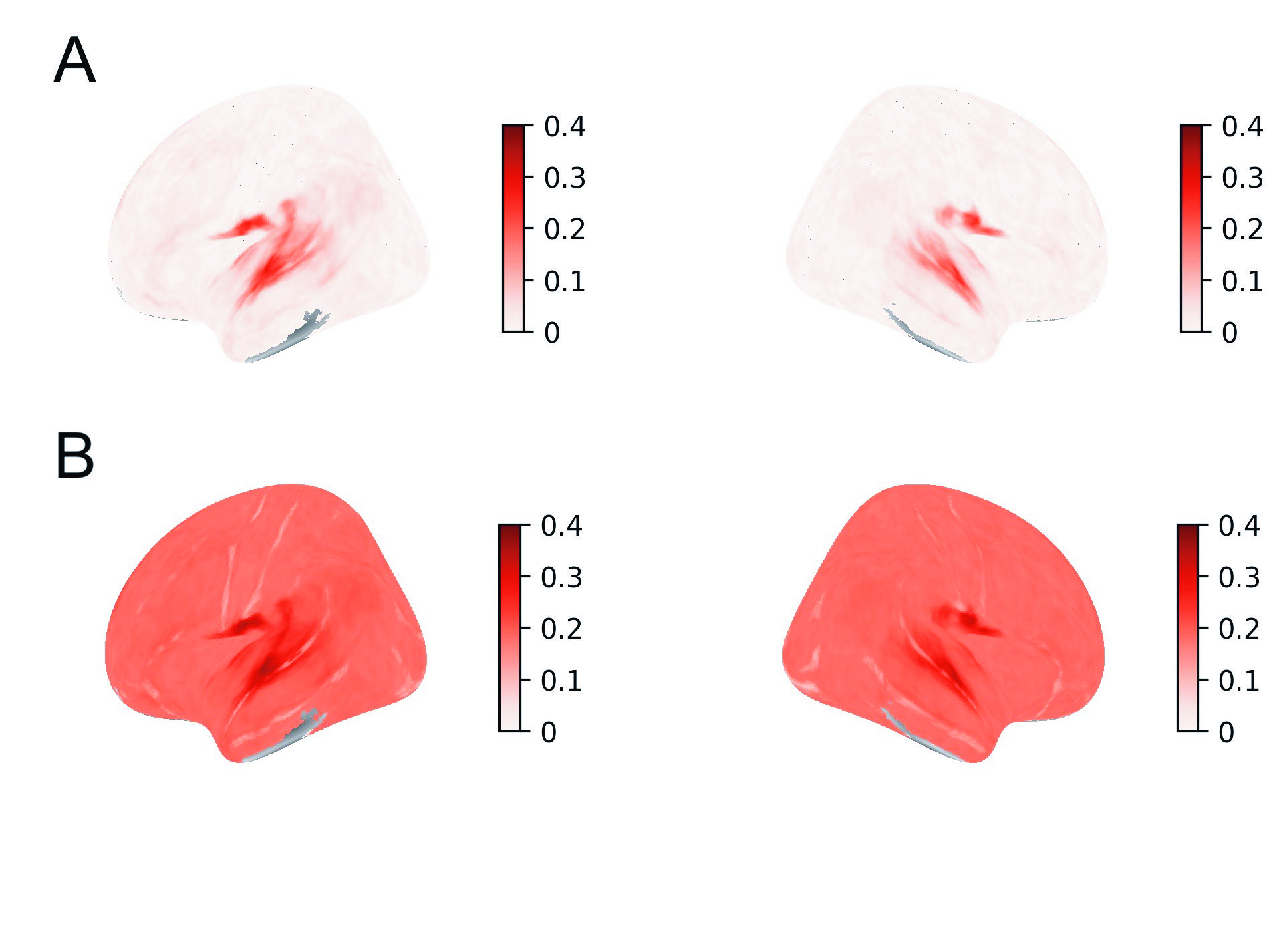

Supplement: nsae032_Supp [file nsae032_supp.zip › sFig10.jpg]

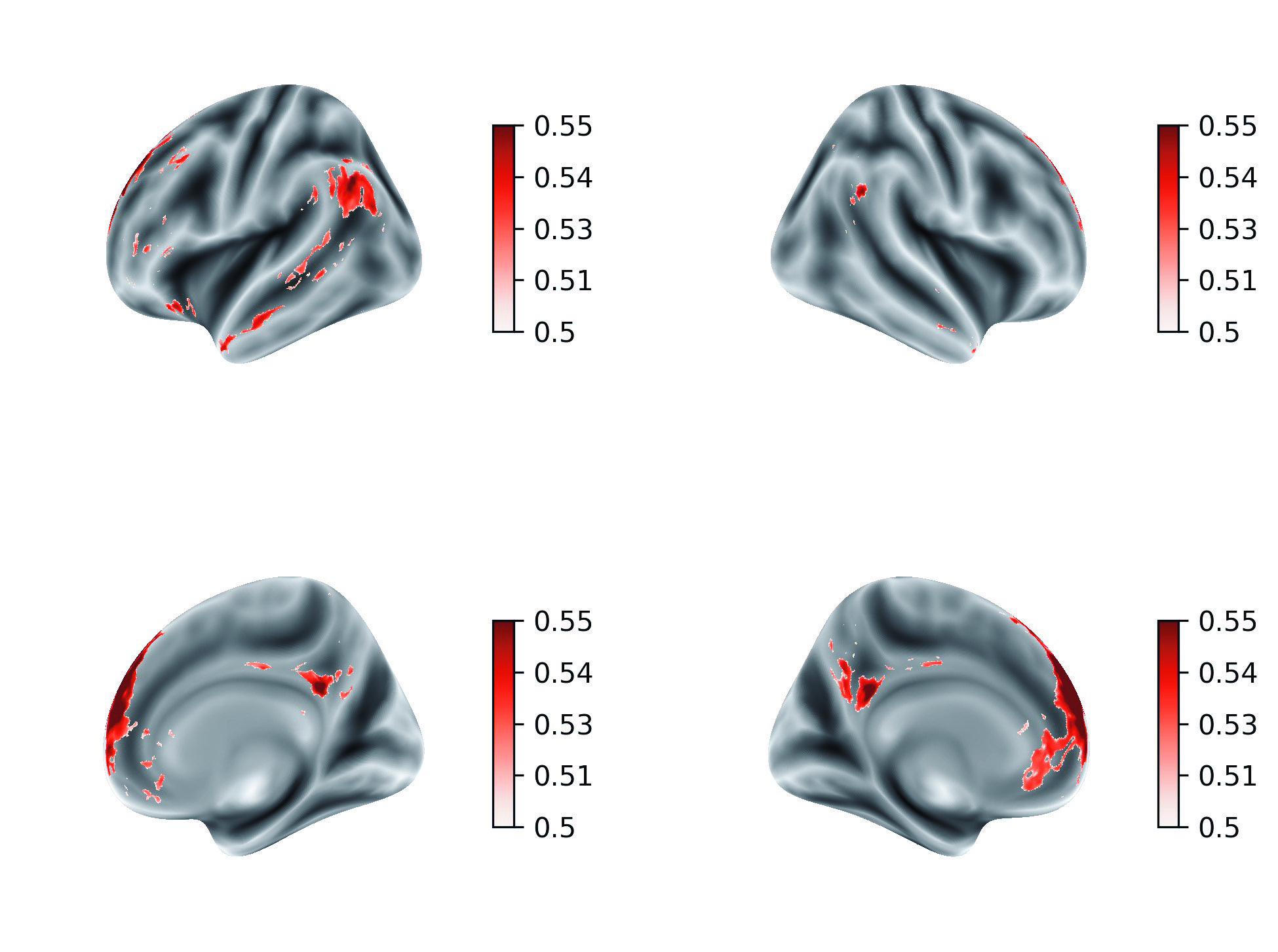

Supplement: nsae032_Supp [file nsae032_supp.zip › sFig2.jpg]

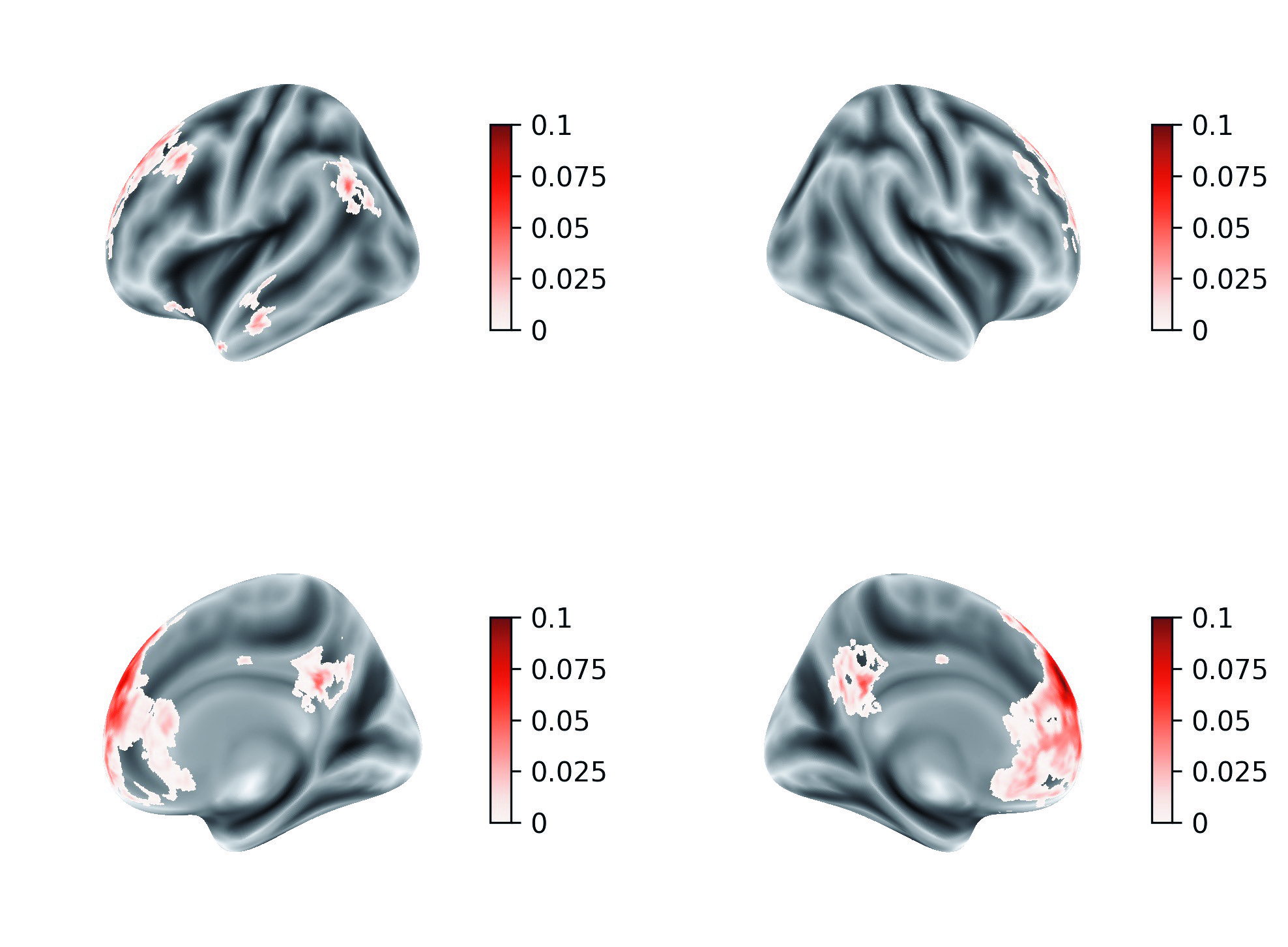

Supplement: nsae032_Supp [file nsae032_supp.zip › sFig3.jpg]

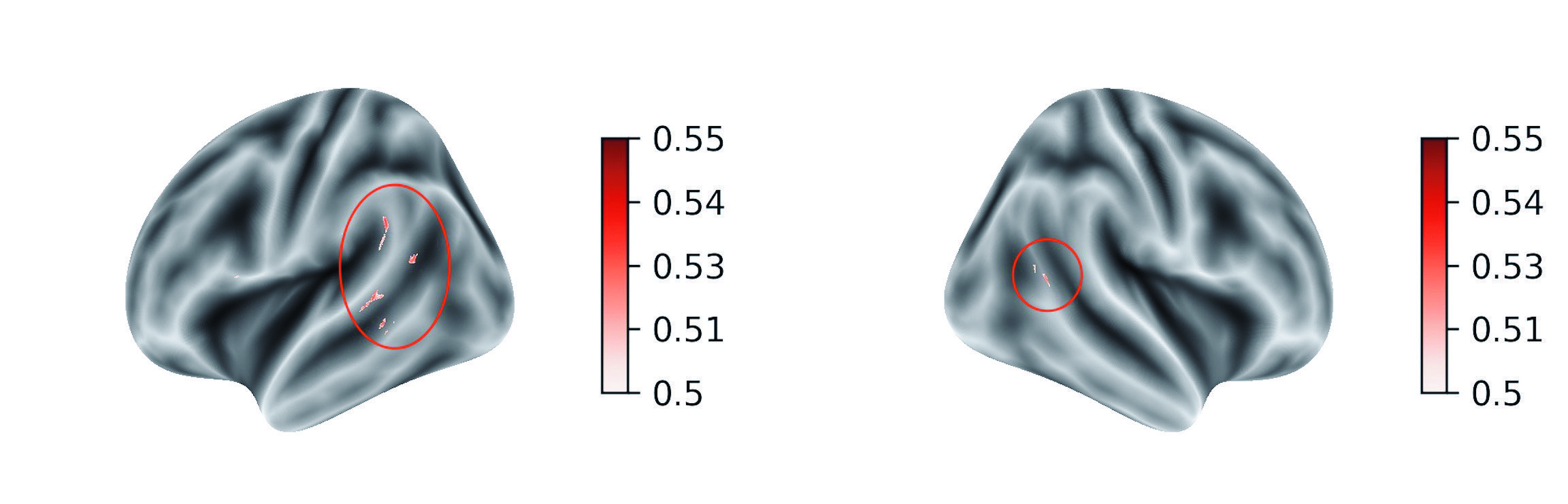

Supplement: nsae032_Supp [file nsae032_supp.zip › sFig4.jpg]

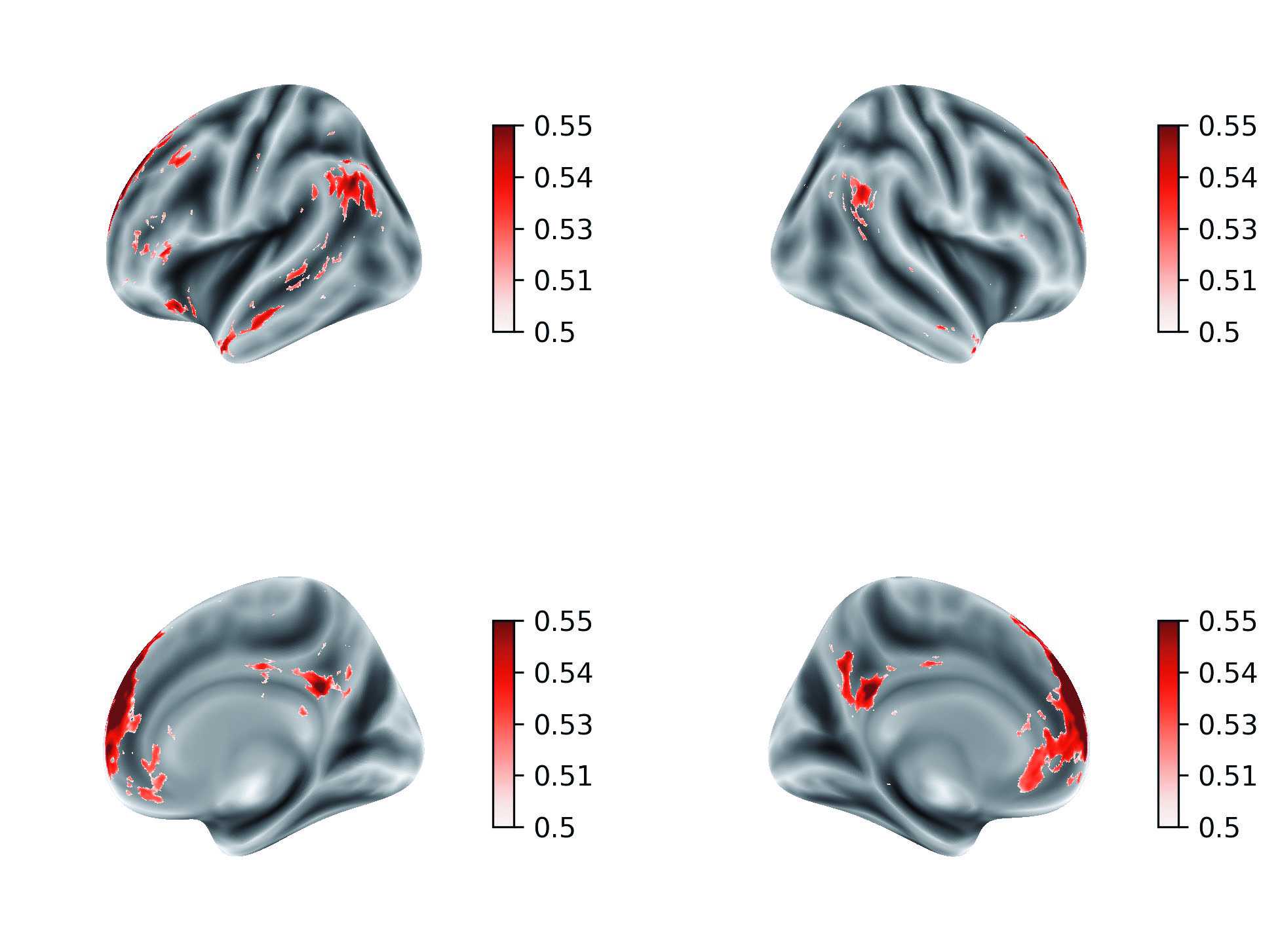

Supplement: nsae032_Supp [file nsae032_supp.zip › sFig5.jpg]

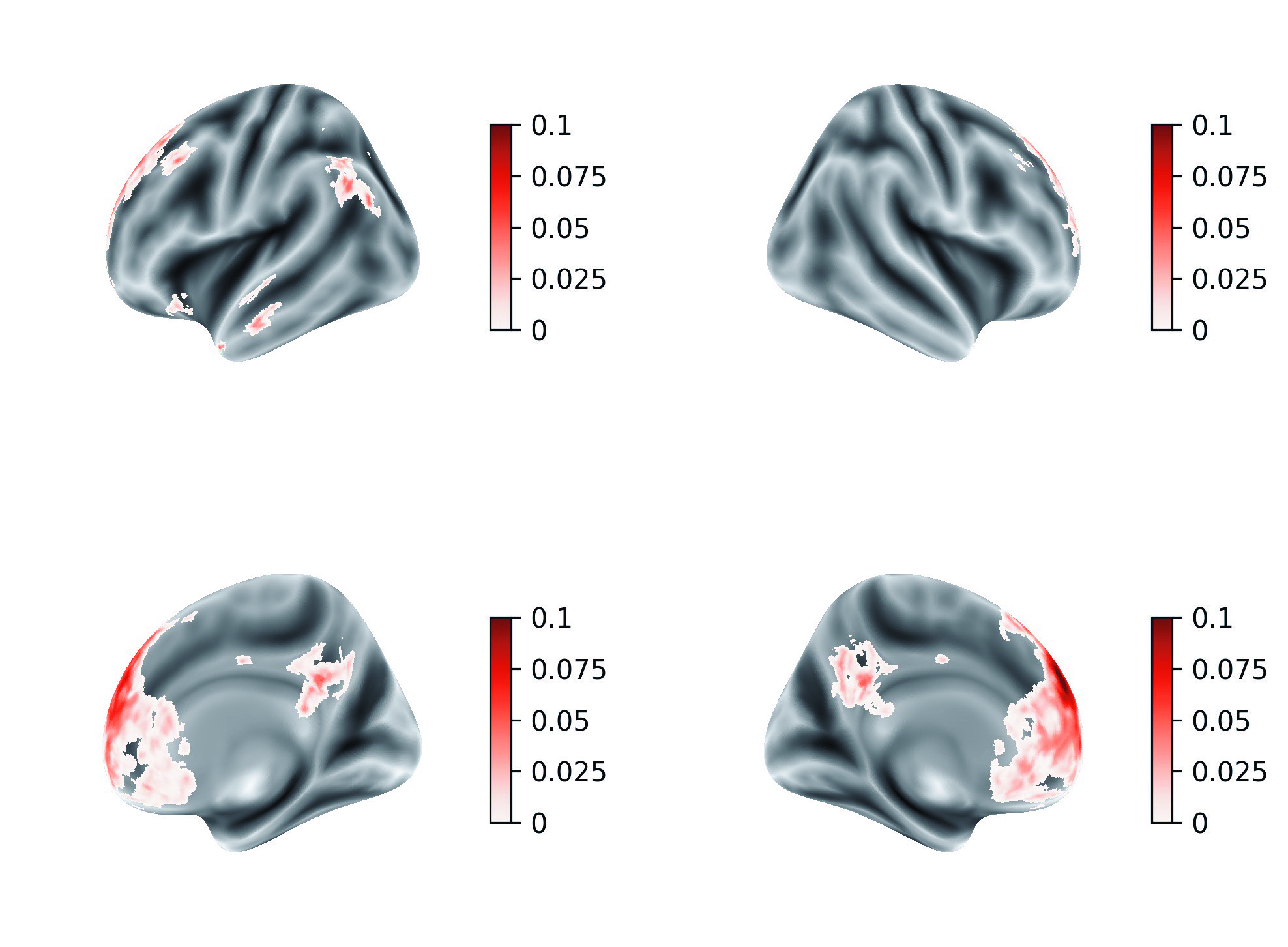

Supplement: nsae032_Supp [file nsae032_supp.zip › sFig6.jpg]

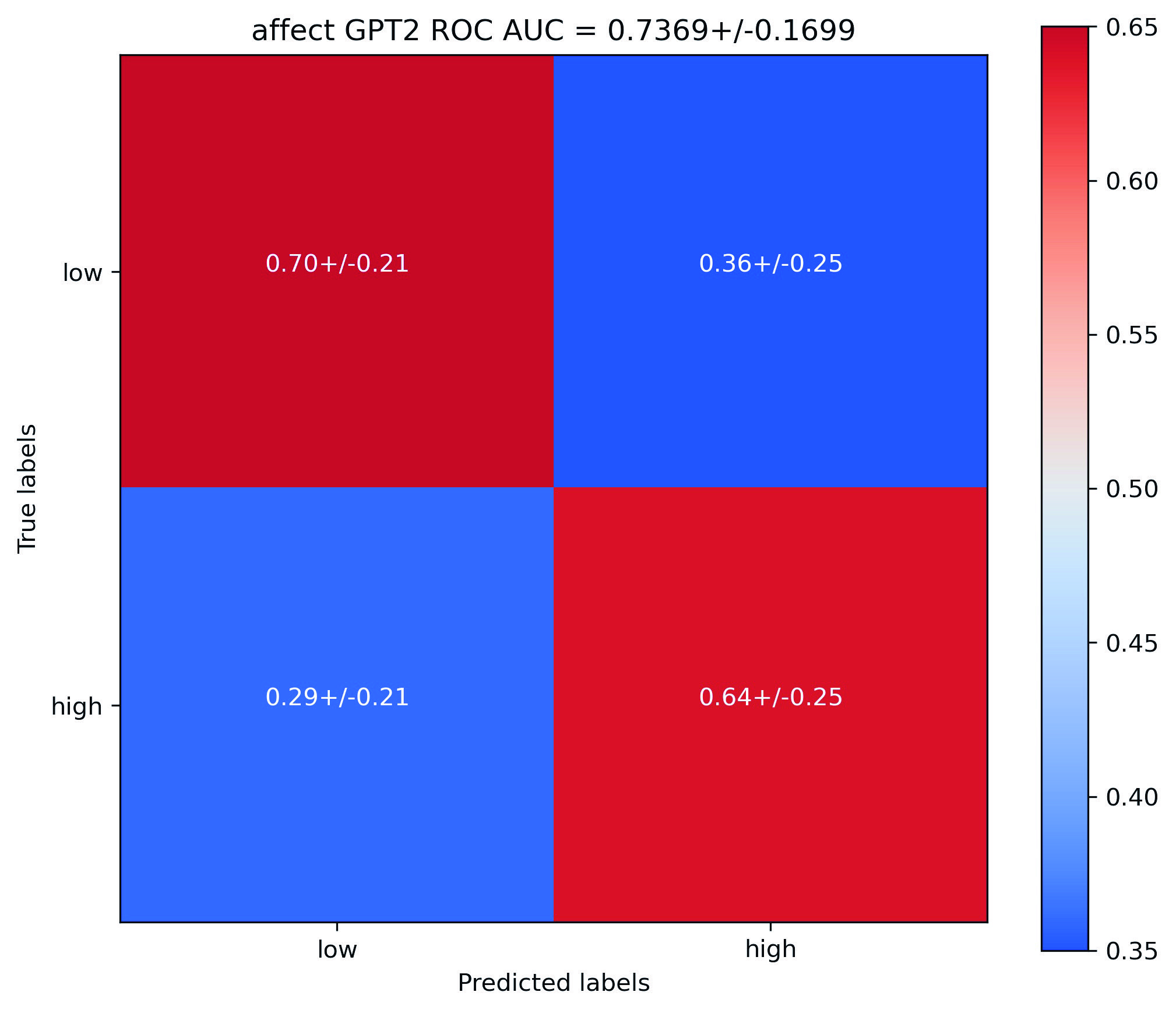

Supplement: nsae032_Supp [file nsae032_supp.zip › sFig7.jpg]

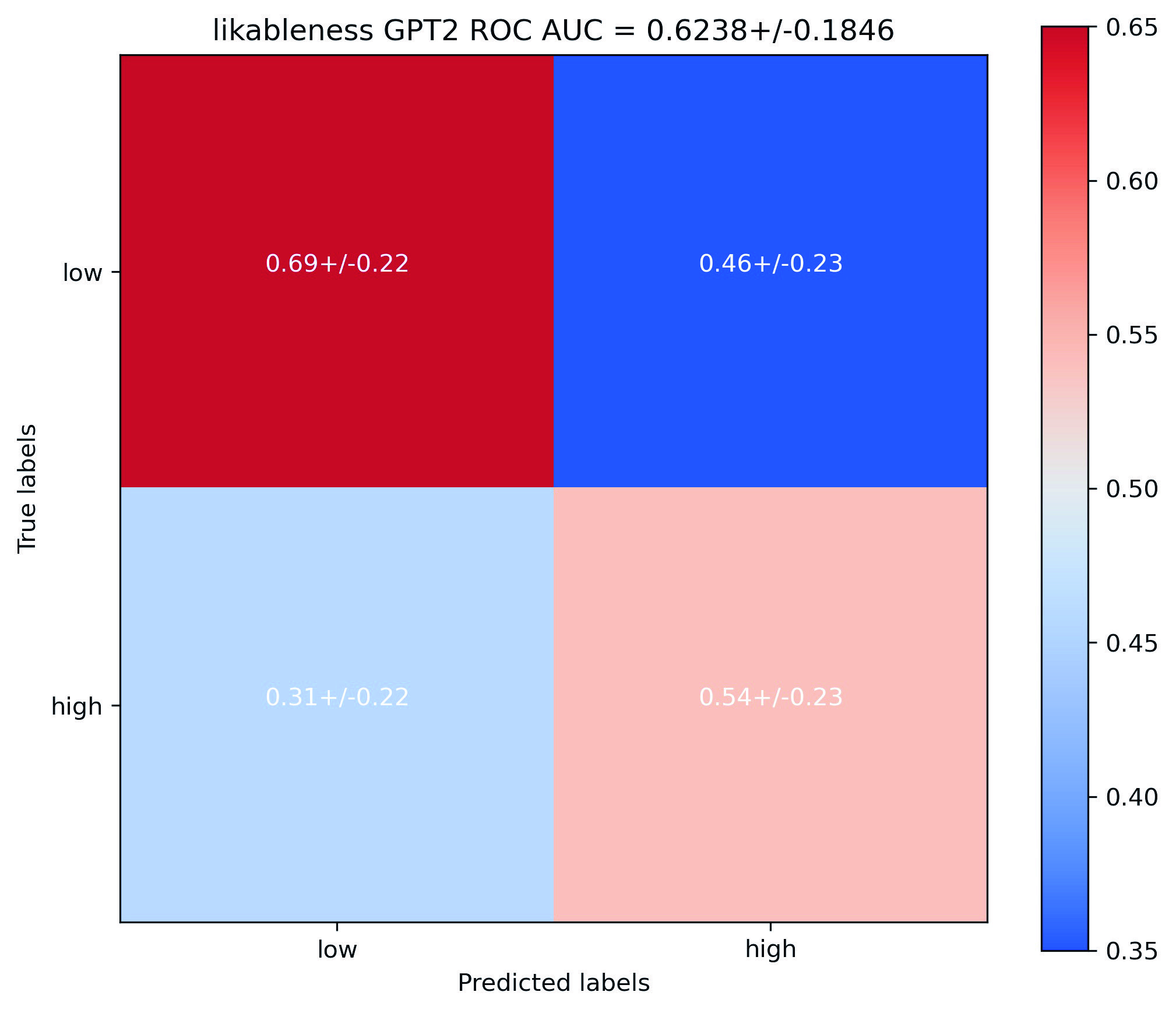

Supplement: nsae032_Supp [file nsae032_supp.zip › sFig8.jpg]

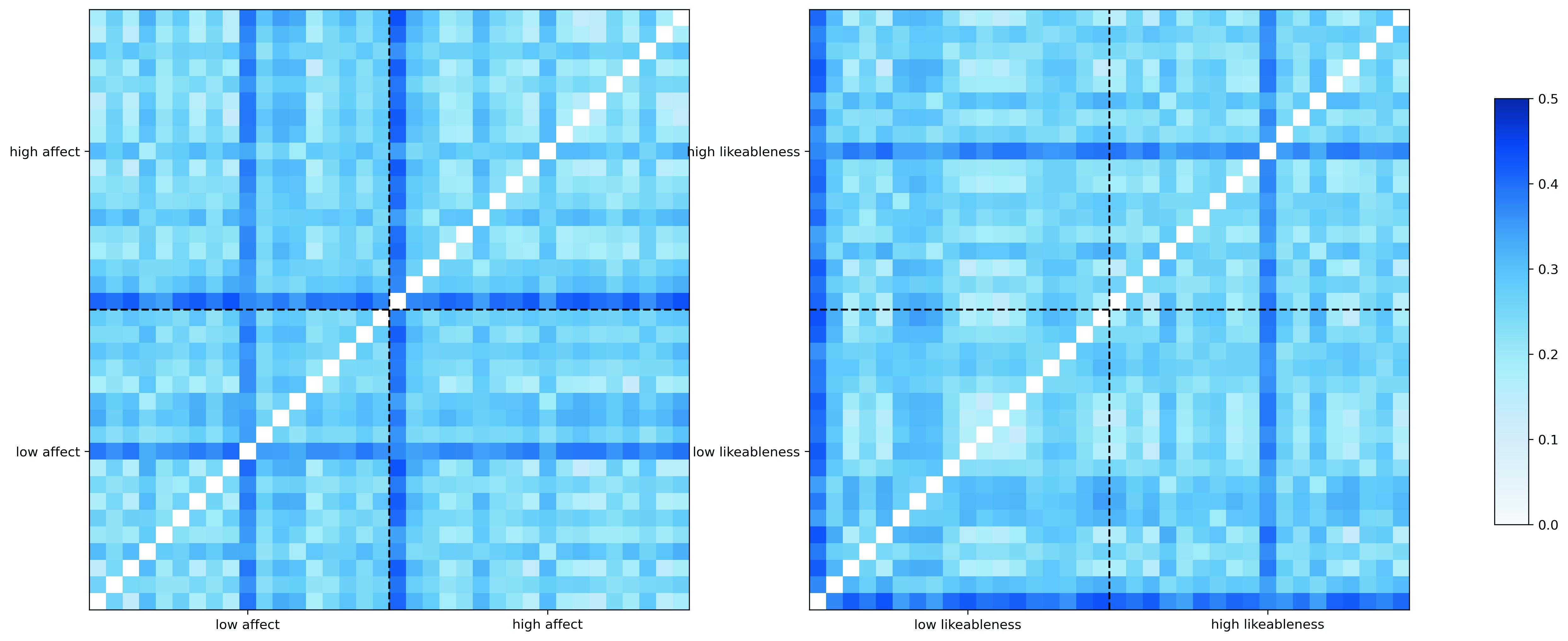

Supplement: nsae032_Supp [file nsae032_supp.zip › sFig9.jpg]
